# Maternal Prenatal Stress, Thyroid Function and Neurodevelopment of the Offspring: A Mini Review of the Literature

**DOI:** 10.3389/fnins.2021.692446

**Published:** 2021-09-08

**Authors:** Foteini Anifantaki, Panagiota Pervanidou, Irene Lambrinoudaki, Konstantinos Panoulis, Nikos Vlahos, Makarios Eleftheriades

**Affiliations:** ^1^Second Department of Obstetrics and Gynaecology, Medical School, National and Kapodistrian University of Athens, Athens, Greece; ^2^First Department of Paediatrics, Medical School, National and Kapodistrian University of Athens, Athens, Greece

**Keywords:** maternal stress, thyroid hormones, glucocorticoids, fetal neurodevelopment, HPT axis, HPA axis

## Abstract

Fetal brain is extremely plastic and vulnerable to environmental influences that may have long-term impact on health and development of the offspring. Both the Hypothalamic-Pituitary-Adrenal (HPA) and the Hypothalamic-Pituitary-Thyroid (HPT) axes are involved in stress responses, whereas, their final effectors, the Glucocorticoids (GCs) and the Thyroid Hormones (TH s), mediate several fundamental processes involved in neurodevelopment. The effects of these hormones on brain development are found to be time and dose-dependent. Regarding THs, the developing fetus depends on maternal supply of hormones, especially in the first half of pregnancy. It is acknowledged that inadequate or excess concentrations of both GCs and THs can separately cause abnormalities in the neuronal and glial structures and functions, with subsequent detrimental effects on postnatal neurocognitive function. Studies are focused on the direct impact of maternal stress and GC excess on growth and neurodevelopment of the offspring. Of particular interest, as results from recent literature data, is building understanding on how chronic stress and alterations of the HPA axis interacts and influences HPT axis and TH production. Animal studies have shown that increased GC concentrations related to maternal stress, most likely reduce maternal and thus fetal circulating THs, either directly or through modifications in the expression of placental enzymes responsible for regulating hormone levels in fetal microenvironment. The purpose of this review is to provide an update on data regarding maternal stress and its impact on fetal neurodevelopment, giving particular emphasis in the interaction of two axes and the subsequent thyroid dysfunction resulting from such circumstances.

## Introduction

Evidence supports that stress during pregnancy may have long-term and detrimental consequences in the development of the offspring. “Maternal prenatal stress” can be defined as any exposure of the expectant mother to stressful life conditions, requiring psychological and physical adaptations. Maternal prenatal stress can be chronic, related to ongoing events, or acute, such as exposure to accidents or other sudden incidents. In addition to exposure to life events, maternal stress can also be related to perinatal mental disorders or physical illnesses and conditions, such as malnutrition, during pregnancy. The assessment of maternal stress varies across studies: stress can be “measured” using questionnaires, clinical interviews, or biomarkers (Pervanidou and Chrousos, 2018; Khambadkone et al., 2020).

Stress can be defined as the condition of threatened homeostasis and it is associated with an activation of the stress system which includes mainly the hypothalamic-pituitary-adrenal (HPA) axis and the sympathetic nervous system (SNS). The stress-induced adaptive neuroendocrine, metabolic, and cardiovascular changes take place both in the central nervous system (CNS) and the periphery of the organism. Centrally, the main mediators of the stress system are the hypothalamic paraventricular nucleus hormones corticotropin-releasing hormone (CRH) and arginine-vasopressin (AVP), the arcuate nucleus pro-opiomelanocortin (POMC)-derived peptides (Chrousos and Gold, 1992) alpha-melanocyte-stimulating hormone and beta-endorphin, and the brainstem norepinephrine (NE) produced in the A1/A2 centers of the arousal-regulating locus coeruleus and the autonomic norepinephrine centers in the brainstem. In the periphery, the end-effectors of the HPA axis are the glucocorticoids (GCs) and those of the SNS are the catecholamines, NE and epinephrine (Chrousos and Gold, 1992; Chrousos, 2009; Qu et al., 2020). In addition to elevations in stress hormones, maternal stress may also be associated with emotional disturbances and unhealthy behaviors, such as depression and anxiety, overeating, smoking, and poor adherence to self-care activities. Both biological and behavioral parameters contribute and affect the fetus and are associated with adverse health outcomes (Pervanidou and Chrousos, 2018).

Chronic stress-related dysregulation of the HPA axis may affect all endocrine systems including gonadal and thyroid axes. Stress hormones can inhibit the aforementioned axes at several levels, whereas estradiol and thyroid hormones (THs) stimulate the stress system (Chrousos, 2009). The activation of the HPA axis is also associated with a reduction in the production of thyroid-stimulating hormone (TSH) and inhibition of the peripheral conversion of thyroxine (T4) to the biologically active triiodothyronine (T3). This may result from the increased concentrations of CRH-induced glucocorticoids and may facilitate the conservation of energy during stress (Benker et al., 1990). In humans, maternal thyroid dysfunction has been associated with child cognitive and motor disabilities, which depicts the importance of THs in neurodevelopment of the fetus and particularly during the first half of gestation, when fetal functions depend primarily on maternal TH supply (Williams, 2008; Miranda and Sousa, 2018).

Fetal life constitutes a critical developmental window, at which, the effects of stress can be transmitted from mother to child. Stress-related maternal, placental and fetal neurobiological alterations may affect the developing fetus, in a process also named “fetal programming” (Barker, 1998). Neurodevelopment of the offspring, the brain’s progressive process affecting the development of neurologic pathways that influence functioning or performance, is an obvious target of research of stress-related alterations during pregnancy. Many factors can affect neurodevelopmental processes in different time and manner during pregnancy and could have a detrimental, unique, and irreversible impact on brain maturation (Stevens et al., 2013; Dowell et al., 2019).

Of particular interest, as accrues from recent literature, is building understanding on how and in which way chronic stress and HPA axis dysfunction interact and influence the hypothalamic-pituitary-thyroid (HPT) axis and TH production. It seems that both axes, as stress-sensitive systems, also located in adjacent regions of CNS, interact with each other, and such relation may affect fetal neurodevelopment (Taylor et al., 1995; Forhead et al., 2007; Moog et al., 2017). The purpose of this review is to provide evidence on the associations between maternal stress and thyroid function and their impact on neurodevelopment.

## Maternal Prenatal Stress, HPA Axis and Fetal Brain Development

Glucocorticoids, the end-effectors of the HPA axis, normally participate in brain maturation and CNS development through structural, molecular, and neurochemical procedures. Studies demonstrate that maternal GCs, that enter fetal circulation through the placenta, are important in several neurodevelopmental processes, such as remodeling axons and dendrites and neuronal and glial cell survival in critical brain regions of the fetus. Any deviation from their normal range of circulating levels is related to abnormalities in cell structure formation, neurotransmission, and function of fetal CNS (Meyer, 1985; Heim et al., 2019; Shallie and Naicker, 2019).

The levels of corticosteroids in fetal circulation, as well as their interaction with particular brain regions, are mediated by specific receptors (mineralocorticoid-receptor, MR-R, glucocorticoid-receptor, GR-R) and enzymes such as 11β- hydroxysteroid dehydrogenase type 2 (11β- HSD2) (Groeneweg et al., 2011; Wyrwoll et al., 2011). The aforementioned degrades GCs to an inactive form and is strongly expressed in fetal brain and placenta (Wyrwoll et al., 2011).

Both clinical and experimental studies, in sheep models and rats, have demonstrated the negative impact of maternal stress and the subsequent GC excess in fetal neurodevelopment. Increased apoptotic activity in the hypothalamus of fetuses whose mothers experienced stress during pregnancy has been observed (Fujioka et al., 1999; Dowell et al., 2019). Alterations in the function of hippocampus, which is involved in regulation of the HPA axis in a feedback process and in cognitive functions linked to memory, have also been detected in cases with an excess of corticosteroids *in utero* (Frodl and O’Keane, 2013). Lemaire et al. (2000) have noticed that prenatal stress leads to a reduction of hippocampal neurogenesis in male rat models. Altered hippocampal neurogenesis and altered glutamatergic hippocampal pathway have been associated with behavioral and emotional disorders in adulthood. In addition to endogenous stress-related glucocorticoid excess, observations in humans have shown that multiple doses of exogenous administration of corticosteroids during pregnancy are associated with significantly lower birth weight, length, and head circumference (Murphy et al., 2008; Green and Nolan, 2014; Pervanidou and Chrousos, 2018).

Animal studies have shown differentiations in hippocampal structure in an environment of elevated levels of GCs. Studies in rats have shown that exposure to high concentrations of GCs leads to an increase of CRH mRNA in the amygdala and a reduction of MR-R and GR-R levels in the hippocampus, thus reducing feedback sensitivity of the HPA axis. In sheep models, excess of betamethasone *in utero* is associated with disturbances in the formation of neurons, myelination, and vascular modulation. Additionally, GC fetal overexposure seems to affect serotonergic and dopaminergic system through a reduction in monoamine oxidase A (MAOA) expression which metabolizes serotonin and dopamine in placenta. The dysregulation of those systems can lead to neurodevelopmental defects and neurobehavioral disorders, including the increased risk of attention-deficit hyperactivity, extrapyramidal disorders and antisocial behaviors such as drug addiction (Uno et al., 1990; Welberg et al., 2000; Wyrwoll and Holmes, 2012).

Moreover, maternal stress and elevated levels of GCs can cause epigenetic alterations which are related to increased cardiovascular tone, reduced insulin sensitivity and increased production of glucose and fat later in life. Recent data address the possibility that environmental events might interfere on DNA methylation, modify the structure of chromatin and thus affect GR gene expression (Meaney et al., 2007).

## Maternal Prenatal Stress and the Placenta

Placental function is critical since it regulates maternal-fetal interactions and participates in fetal homeostasis and development (Garnica and Chan, 1996). One of its main actions is to control fetal exposure to maternal cortisol excess via the expression of the enzyme 11β- HSD2. As mentioned above, 11β-HSD2 enzyme normally converts active cortisol into inactive cortisone and eventually acts as a barrier preventing the transfer of elevated levels of GCs from maternal to fetal circulation (Howerton and Bale, 2012; Glover et al., 2018; Zhang et al., 2018). Maternal secretion of glucocorticoids changes profoundly during pregnancy with circulating cortisol levels rising approximately threefold by delivery (Jung et al., 2011). It has been observed that the enzyme is highly expressed during the second and third trimesters of pregnancy in humans (Glover et al., 2018).

Studies in humans, as well as in rodents, have shown that maternal stress, related to anxiety, depression, or other unfavorable psychological conditions, downregulates the expression and activity of 11β-HSD2 formatting a fetal environment with abnormally elevated levels of GCs (O’Donnell et al., 2012; Monk et al., 2016; Glover et al., 2018). Furthermore, severe obstetric complications such as fetal growth restriction (FGR) and preeclampsia have been associated with decreased 11β-HSD2 activity and dysfunction, respectively (Zhang et al., 2018; Wang et al., 2020).

Other observations, concerning the impact of maternal stress on the placenta and its critical endocrine functions, include an interruption in the production of neurotransmitters and neurotrophins that play key roles in fetal neurodevelopment (Gur et al., 2017). Studies in rodent models have shown an increase in placental production of proinflammatory cytokines interleukin-6, interleukin-1β (IL-6, IL-1β), and tumor necrosis factor-a (TNF-a), which influences normal developmental processes of the fetus (Bronson and Bale, 2014; Dowell et al., 2019). Zhang et al. (2018) studied women who have been exposed to acute stress during a natural disaster and have demonstrated a reduction in the expression of specific placental genes (HSD11B2, MADA, and ZNF507). Finally, maternal stress seems to be associated with placental hypoxia due to restricted umbilical artery flow which consequently interrupts normal neuronal migration and myelination (Sjöström et al., 1997).

## HPT Axis, THs, and Fetal Brain Development

The parvocellular region of the paraventricular nucleus of hypothalamus (PVN) is responsible for the synthesis and secretion of thyrotropin-releasing hormone (TRH), which consequently stimulates the release of TSH from the anterior pituitary (Bernal, 2007). However, the largest concentrations of hypothalamic TRH neurons outside of the PVN are found in the dorsomedial nucleus, lateral hypothalamus and preoptic area, including medial, periventricular, suprachiasmatic and the sexual dimorphic nucleus of the preoptic area (Nillni, 2010). The regulation of circulating levels of THs, as well as their intracellular transport in specific organs and tissues, is mediated by specific transmembrane transporters, including members of the organic anion transporting peptide family (OATPs), nuclear receptors and enzymes (Visser et al., 2008). The action of THs in the brain is mainly mediated by nuclear TH receptors that bind T3 and subsequently lead to the expression of genes that participate in neurodevelopmental procedures. These receptors are found principally in oligodendrocytes and neurons as early as 10 weeks of gestation (Bernal, 2007; Miranda and Sousa, 2018).

The role of the three types of iodothyronine deiodinases (D1, D2, and D3) that are expressed in different organ tissues and are responsible for the activation or inactivation of T4 and T3 is of great importance. Specifically, D1 is responsible for the large fraction of the circulating T3, by converting T4 to T3 and is expressed in multiple organs including placenta. D2, which is also active in placenta, participates in the local production of approximately 80% of CNS T3 from T4. Placenta mainly express D3, which is also present in brain tissue and blocks the action of T3 by restricting the transport of maternal THs to fetal circulation (Crantz et al., 1982; Moog et al., 2017).

The fetus collects iodine and synthesizes THs by 11–12 weeks of gestation, while the secretion of THs begins around week 16. Although fetal thyroid gland is capable of TH production, it is not fully matured during intrauterine life, thus maternal transfer of THs through the placenta occurs until birth and is responsible for normal TH levels in fetal circulation (Morreale de Escobar et al., 2004). THs are detected in the human cerebral cortex by 12 weeks of gestation, and even earlier, in other brain structures. Thus, maternal TH levels, which also depict fetal TH levels during the first half of pregnancy, need to remain within strict levels to assure normal neurodevelopment (Miranda and Sousa, 2018).

THs are of significant importance in maturation and functional formation of the brain throughout all trimesters of pregnancy, participating in neurodevelopmental procedures including neuron proliferation and migration, neuronal and glial cell differentiation, and synaptogenesis. TH level disruptions can also alter biochemistry of the brain. Animal studies have shown that an environment of hypothyroidism is related to a decrease in monoamines, such as epinephrine and NE, and a concurrent increase in γ-aminobutyric acid (GABA) (Ahmed et al., 2010; Moog et al., 2017). GABA is a neurotransmitter that plays important role in early neurodevelopment of the embryo as it acts as a trophic factor for processes such as cell proliferation and migration (Ahmed et al., 2010). Myelination of the axons in CNS is strongly TH-dependent as THs affect the expression of genes that are responsible for the production of myelin proteins (proteolipid protein, myelin basic protein, and myelin-associated glycoprotein) (Sutcliffe, 1988).

TH deficiency, especially during the early critical embryonic stages of development, has an impact not only in molecular and functional pathways of the brain, but also in morphology of brain regions such as cerebral cortex, hippocampus, and cerebellum (Gould et al., 1991; El-Bakry et al., 2010; Shallie and Naicker, 2019). Additional abnormalities that have been observed and can be attributed to maternal hypothyroidism include limited growth of axons and dendrites, decreased and abnormal neuronal connectivity, myelin deficits, and reduction in synaptic densities (Opazo et al., 2008). On the contrary, hyperthyroidism appears to accelerate neurodevelopmental procedures which lead to premature proliferation and cell differentiation. Such circumstances seem to establish a shorter average length of cell cycle which is reflected in a decreased weight of brain tissue and body, degenerations in brain regions and premature termination of cerebellar processes (Shallie and Naicker, 2019).

## Interaction of HPA and HPT Axes

Animal and human studies have demonstrated that HPT axis is also a stress-sensitive system that interacts with the HPA axis in multiple ways (Supplementary Figure 1). Both axes communicate anatomically in the level of the hypothalamus and pituitary gland. Subsequently, due to their adjacency, dysregulation of either axis is likely to cause imbalance to the other (Moog et al., 2017).

Exogenous administration of GCs in humans has an inhibitory effect on thyroid function which is depicted in decreased plasma TSH levels, low response of TSH to TRH stimulation, and an enhancement of the negative feedback of T3 on TSH release. Regarding THs, elevated levels of GCs may lead to a reduction in T3 and an increase in reverse T3 (rT3) levels, while T4 may be unchanged or slightly decreased (Bános et al., 1979). Other actions of GCs on TH metabolism that have been proposed include iodine metabolism modifications, inhibition of the secretory activity of the thyroid gland and differentiation in deiodinase activity or TH receptor expression (Miranda and Sousa, 2018; Shallie and Naicker, 2019).

Depression, and any other condition which is related to maternal stress and increased cortisol levels, most likely lead to a reduction of maternal circulating THs, meaning that lower levels of maternal T4 cross the placenta and reach fetal circulation, and consequently fetal brain (Bauer et al., 1994). Regarding fetal neurodevelopment, it has been shown in sheep models that maternal and fetal administration of glucocorticoids in the third trimester of pregnancy increases plasma T3 and rT3 but not T4 concentrations. One mechanism that probably is responsible for the modifications in fetal TH metabolism is the reduction of placental D3 and the concurrent increase in fetal D1 activity (Forhead et al., 2007). It must be mentioned that the vast majority of T3 in fetal brain originates from local conversion of T4 to T3 by D2 effect. Additionally, due to the difference in activity levels of iodothyronine deiodinases among trimesters, the impact of TH concentrations in early pregnancy may be different from that shown in term (Crantz et al., 1982).

Diversely, HPT dysfunction can affect HPA axis and influence adenocorticotropic hormone (ACTH) and GC production. High levels of THs accelerate HPA axis maturation by inducing hypersensitivity of cortisol to ACTH (Lizcano and Rodríguez, 2011). Furthermore, low circulated THs seem to decrease cortisol blood clearance and impair 11β-HSD2 activity (Brown et al., 1958). Many different mechanisms have been described in the literature, but these data are beyond the scope of this review. In conclusion, GCs and THs seem to have a synergistic effect on fetal brain maturation and neurodevelopment and both their concentrations must be adequate and within the normal range in fetal circulation.

## Discussion

Neurodevelopment and maturation of the brain is a complicated process that begins in fetal life and continues the years after birth. Production and circulated levels of GCs and THs depict the normal function of two important stress-sensitive axes, the HPA and HPT respectively (Supplementary Figure 2). Such hormones are involved in important processes for embryonic neurodevelopment, including neuron proliferation, neuron migration, and differentiation as well as myelination and seem to act not only independently but also synergically through complex and not completely understood pathways (Bános et al., 1979; Bernal, 2007; Khulan and Drake, 2012).

It is acknowledged that abnormal concentrations of both GCs and THs can separately affect the neuronal and glial structures, as well as brain functions with a subsequent negative impact on cognition in later life. Specific brain structures including hippocampus, which is implicated in cognitive processes such as memory and learning, are more vulnerable to such modifications and could lead to long-term developmental implications for the offspring (Gould et al., 1991).

Maternal stress has been related to the activation of the HPA axis and secretion of greater amounts of glucocorticoids that enter fetal circulation and affect fetal HPA axis development and fetal GC levels. The aforementioned mechanism can lead to severe deviation from normal neurodevelopmental procedures (Glover et al., 2018). Stress has also been associated with defects in fetal growth and reduced birthweight, through alterations in amounts of nutrients and important substances that cross the placenta (Dowell et al., 2019; Troller-Renfree et al., 2020). Studies confirm that hyperactivation of HPA axis has detrimental effects on reproductive, growth, thyroid and immune functions and may lead to metabolic, cardiovascular and neuroendocrine disorders in adulthood, such as hypertension, diabetes and metabolic syndrome. Additionally, a link between GCs excess, due to maternal stress, and mood disorders, including depression, anxiety as wells as disturbances in circadian rhythmicity has also been proposed (Maccari and Morley-Fletcher, 2007).

The placenta consists another target organ involved in maternal stress reactions, and several functional changes have been observed under stressful conditions. Modifications in the expression of critical placental substances, such as enzyme 11β-HSD2, contribute to the abnormal circulating hormone levels in the fetal environment (O’Donnell et al., 2012).

THs play a key role in the early development of the fetus, the placenta, and the differentiation of fetal developing tissues including the brain. It is well known that congenital hypothyroidism is related to severe neurological impairment and intellectual disability. Other thyroid dysfunctions, including maternal hypothyroxinemia due to iodine deficiency, are also risk factors for neurodevelopmental handicaps, particularly during the first half of pregnancy (Chen and Hetzel, 2010). As mentioned above stress impairs maternal and subsequent fetal thyroid function. It has been observed that HPT axis reacts differently in each type of stress. Specifically, acute, and chronic stress decrease peripheral TH levels whereas traumatic stress may activate thyroid and can lead to thyrotoxicosis (Friedman et al., 1999).

Multiple and different mechanisms through which THs participate in fetal neurological structure formation and function have been described, with some of them still not be fully understood. However, there is enough data to support the necessity of adequate supplied amounts of such hormones to ensure optimal neurodevelopment throughout ontogeny (Shallie and Naicker, 2019).

Of particular interest is building an understanding on how chronic stress and HPA axis interact and influence HPT axis and TH production. Animal studies have shown that increased GC concentrations related to maternal stress, most likely reduce maternal TH levels, thus lower levels of THs reach fetal circulation. Interaction of the two axes seems to be direct, as well as indirect, through modifications in the expression of specific enzymes responsible for regulating TH levels in fetal microenvironment. From the above data, it can be hypothesized that chronic stress and activation of HPA axis seem to influence TH production through a premature maturation of fetal HPT axis (Miranda and Sousa, 2018).

As published evidence is derived from heterogenous studies, regarding design and scope, further targeted research is needed to better understand the influence of gestational stress and HPA axis dysregulation on thyroid gland function. Given the association of thyroid dysfunction with maternal stress, pregnant women would be an interesting target of research. This would help to improve neurodevelopmental outcomes, and to prevent cognitive, behavioral and mental disorders related to maternal thyroid dysfunction later in life.

## Author Contributions

FA and ME contributed to conception of the review and also wrote the first draft of the manuscript. PP, IL, KP, and NV contributed to manuscript revision, read, and approved the submitted version. All authors contributed to the article and approved the submitted version.

## Conflict of Interest

The authors declare that the research was conducted in the absence of any commercial or financial relationships that could be construed as a potential conflict of interest.

## Publisher’s Note

All claims expressed in this article are solely those of the authors and do not necessarily represent those of their affiliated organizations, or those of the publisher, the editors and the reviewers. Any product that may be evaluated in this article, or claim that may be made by its manufacturer, is not guaranteed or endorsed by the publisher.

## Supplementary Material

Supplementary Figure 1 Interactions of HPA and HPT axes. Solid lines represent activation, and dashed lines represent inhibition. ACTH, corticotropin; CRH, corticotropin-releasing hormone; TRH, thyrotropin-releasing hormone; TSH, thyroid-stimulating hormone.

Supplementary Figure 2 Prenatal Maternal Stress is related with biological and behavioral changes that can affect fetal brain development. Thyroid Gland has independent effects on neurodevelopment but also interacts with the stress system in a reciprocal manner. CRH, corticotropin-releasing hormone; 11beta- HSD2, 11beta- hydroxysteroid dehydrogenase type 2; HPA, Hypothalamic-Pituitary-Adrenal, SNS, Sympathetic Nervous System; THs, Thyroid Hormones; IL-6, Interleukin-6.
