# Supplementary material for: Maternal Prenatal Stress, Thyroid Function and Neurodevelopment of the Offspring: A Mini Review of the Literature
Source: Front Neurosci. 2021 Sep 8;15:692446. doi: 10.3389/fnins.2021.692446 (PMC8455916; doi:10.3389/fnins.2021.692446)
Supplement: Supplementary Figure 1 — Interactions of HPA and HPT axes. Solid lines represent activation, and dashed lines represent inhibition. ACTH, corticotropin; CRH, corticotropin-releasing hormone; TRH, thyrotropin-releasing hormone; TSH, thyroid-stimulating hormone. [file Image_1.pdf]

Supplementary Material

1 Figure

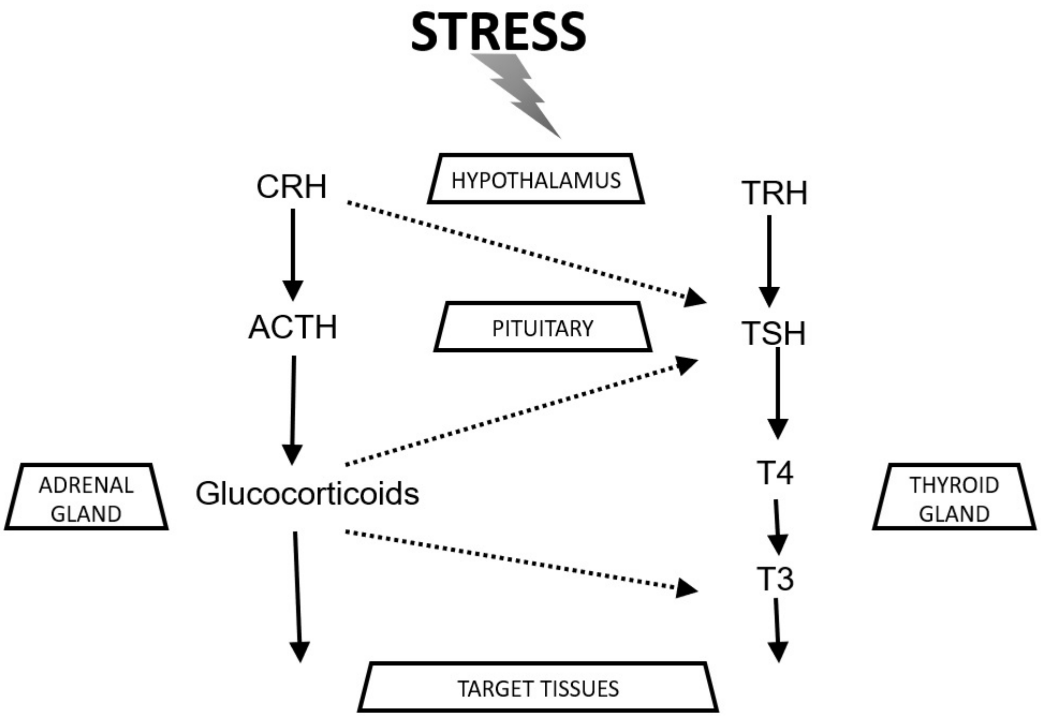

Interactions of HPA and thyroid axes.
Solid lines represent activation, and dashed lines represent inhibition
Abbreviations: ACTH = corticotropin; CRH = corticotropin-releasing hormone; TRH = thyrotropin-releasing hormone; TSH = thyroid-stimulating hormone
