# Supplementary material for: Maternal Prenatal Stress, Thyroid Function and Neurodevelopment of the Offspring: A Mini Review of the Literature
Source: Front Neurosci. 2021 Sep 8;15:692446. doi: 10.3389/fnins.2021.692446 (PMC8455916; doi:10.3389/fnins.2021.692446)
Supplement: Supplementary Figure 2 — Prenatal Maternal Stress is related with biological and behavioral changes that can affect fetal brain development. Thyroid Gland has independent effects on neurodevelopment but also interacts with the stress system in a reciprocal manner. CRH, corticotropin-releasing hormone; 11beta- HSD2, 11beta- hydroxysteroid dehydrogenase type 2; HPA, Hypothalamic-Pituitary-Adrenal, SNS, Sympathetic Nervous System; THs, Thyroid Hormones; IL-6, Interleukin-6. [file Image_2.pdf]

Supplementary Material

2 Figure

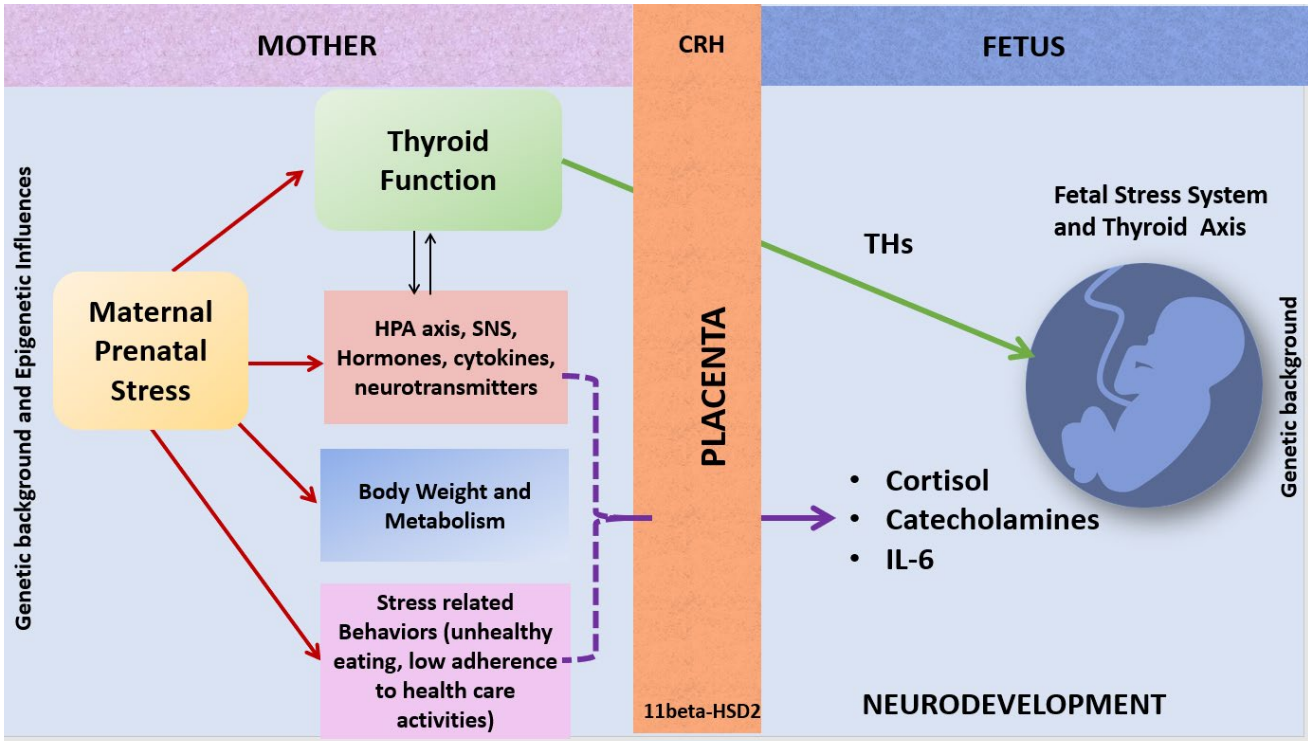

Prenatal Maternal Stress is related with biological and behavioral changes that can affect fetal brain development. Thyroid Gland has independent effects on neurodevelopment but also interacts with the stress system in a reciprocal manner.

Abbreviations: CRH: corticotropin-releasing hormone, 11beta- HSD2: 11beta- hydroxysteroid dehydrogenase type 2, HPA: Hypothalamic-Pituitary-Adrenal, SNS: Sympathetic Nervous System, THs: Thyroid Hormones, IL-6: Interleukin-6
